# A Bold Declaration Reviewed

**Published:** 1887-02

**Authors:** C. A. Marvin


### Independent Practitioner.

Vol. VIII. February, 1887. No. 2.

### Original Communications.

Note.—No paper published or to be published in another journal will be accepted for this department. All papers must be in the hands of the Editor before the first day of the month preceding that in which they are expected to appear. Extra copies will be furnished to each contributor of an accepted original article, and reprints, in pamphlet form, may be had at the cost of the paper, press-work and binding, if ordered when the manuscript is forwarded. The Editor and Publishers are not responsible for the opinions expressed by contributors. The journal is issued promptly, on the first day of each month.

### A BOLD DECLARATION REVIEWED.

BY DR. C. A. MARVIN.

Read before the Second District Dental Society at its Meeting in Brooklyn, January 10, 1887.

Without prefixing any formal title to the paper I am about to present, I ask your attention while I consider from my standpoint —which I hope to be able to show is the correct standpoint—the startling declaration recently made by a member of our profession, that "dentistry is not a specialty of medicine."

This declaration is accompanied by a lengthy, exhaustive argument, supposed to be convincing to all minds capable of weighing argument and recognizing truth.

The whole—declaration and argument—comes to us in the shape of a formal paper read before several dental societies, published, in synopsis, in a medical journal, and *in extenso* in a dental journal, and asking endorsement, immediate and unreserved, from the societies, in the form of an affirmative vote upon some previously prepared resolutions, to be offered when the paper had been read. We are also told that such resolutions have actually been passed by two or more dental societies.

If this is so, and resolutions of endorsement have been affirmatively voted with a haste so unseemly, I can only conclude that, among mental narcotics, a novel and radical assertion, clothed in smooth and apparently ingenuous language, is henceforth to take first rank, its stupefying influence being proportionate to its boldness of statement and disregard of commonly accepted theories. That gentlemen of judgment, of experience, of years, should consent by a formal vote to endorse any such radical proposition immediately upon its presentation, without time for calm consideration, is almost as surprising as the assurance of the utterance itself.

One word more of preface: It is to be regretted that the State Dental Society is apparently committed to the position taken in the paper. I say *apparently,* not really. If the author had been content with signing his name, and that only, the paper would have been known as the expression of his individual views, which, in fact, it is. But when the utterance is given to the public as from the "President of the New York State Dental Society," which State Society has not authorized any such utterance nor had knowledge of any such intention, a false impression is given, unfair to that society, and not justified by any action it has ever taken. Any weight which the name of an important society might constructively add to the published views of an individual officer, is immediately lost when it comes to be understood that any such use of its name was wholly unwarranted.

The paper which I am to review, *as a paper,* I consider excellent. It presents many valuable ideas, culls from the large field of dentistry many facts differing from each other, but each one characterizing some particular section of that large field, advances many suggestions, and opens the way to many lines of thought at once interesting, instructive and pertinent to dental inquiry. I find myself agreeing, as I doubt not a majority of the profession will at once agree, with the statement of the complex nature of dentistry (it has been stated many times before), of the necessity in order to be a competent dentist for artistic talent, mechanical talent, indeed, in the language of the author, of some knowledge "not only of nearly all the sciences, but in an equal degree nearly all the arts." This last is a sweeping sentence, but I do not make a face in the attempt to swallow even this. Indeed, without taking time to particularize, the paper is rich in good thoughts, and those thoughts, as is always the case with the author under consideration, are well and forcibly expressed. It is refreshing to read or hear a paper which distinctly states what its author means, without ambiguity or circumlocution. We then know just what we are asked to accept.

While, as I have said, I agree with many, nay most of the ideas advanced respecting dentistry as a calling, I do not agree with the proposition contained in the title of the paper, nor admit the force of the arguments brought to its support. Had that title been, Dentistry *more* than a Specialty of Medicine, I should have said "amen," and so would any sensible man. But dentistry in no sense a specialty of medicine? Oh, no! I cannot agree to that. The daughter may have arrayed herself so differently, may have acquired accomplishments so distinct, may have taken on habits of living, of activity so diverse that she can hardly be recognized; but let her not deny her parentage. Let her not refuse to credit to that parentage the fundamental rules of conduct and principles of life which have formed the basis of her subsequently erected character, and which she finds herself necessarily employing every day, even in her supposed new and different sphere of action. To deny her kinship is to cast away an honorable connection and become a nameless waif. Nay, more; it bespeaks an ignoble mind and betrays an unworthy ambition. Besides all this, it is not true.

The illustration of "the three tailors of Tooley St." can, without violence, be transferred to the other side of the argument. Suppose they had passed a different resolution, to-wit: "Resolved, that we do *not* belong to the 'People of England,' but are an independent nation ourselves," would they have displayed any more wisdom or been any the less Englishmen? To ask the question is to answer it.

In commencing the task before me I first inquire, what is the aim of dentistry? and quoting from the author under consideration, "by dentistry I mean to include every branch and department known under that name, and a dentist in the full sense of the term

* * * is one who understands and can practice each and every specialty of it." With this definition before us, I ask again, what is the aim of dentistry?

To this question I desire an answer that shall embody the truest and loftiest sentiment of the best men in it. To afford a livelihood to him who has chosen it as his vocation? To make money? To achieve fame? To exhibit the possibilities of special science? All these are answers involving a modicum of truth, but none come up to the level of what is required. Here is another: To conserve the well-being of humanity. But this is the aim also of Christian philanthropists. Something more explicit is necessary.

First and highest of all, the true aim of dentistry is to relieve human suffering and promote human health. An aching tooth causes suffering as does an aching head. Unrelieved suffering impairs the health. An alveolar abscess causes suffering as does an auricular abscess. If not relieved, the former will sometimes cause death, and the latter the loss of the sense of hearing. Decayed teeth interfere seriously with perfect mastication Imperfectly masticated food is a prime cause of indigestion. Dyspepsia with its long train of attendant ills ensues, and life is made miserable. The loss of the teeth renders mastication impossible, and necessitates the use of liquid or farinaceous foods, with insufficient nutrition as a frequent result, hence an impaired condition of health.

Pyorrhœa alveolaris makes the mouth sore, unclean, and by its pus secretions mixing with food, unhealthy.

Calcareous deposits secondarily, and lodged *residua* of dissolved food, partially decomposed, primarily, work an unhealthy condition of the mouth, manifested by foul breath, by turgid gums, by incipient or developed epulis. Result, impaired health.

Now, every one of these several vitiated conditions of the mouth is considered a specially appropriate field for a dentist. It is a dentist's legitimate work, generally recognized and admitted. I have been careful to include in this category none of the diseases of the oral cavity, whose legitimate assignment to the care of the dentist is questioned. This being so, a single glance will suffice to render the point I make clear to my hearers. A dentist is called to attend to each and all of these diseased conditions. Now note well his action.

In the case of an aching tooth, he applies a remedy for the express purpose of relieving the pain. The natural result of such relief is an improvement in health. In the case of an alveolar abscess, the treatment is directed to the special end of curing the disease and, as far as possible, of preventing its recurrence. It is not necessary to say that the condition of health is inseparably connected with the local difficulty and dependent upon the success of the dentist's treatment of it.

Nor is the ultimate aim any different when decayed teeth are to be filled, lost teeth superseded by artificial ones, diseases of the gums to be treated, calcareous deposits to be removed, or wrecked teeth to be extracted. These are only varying phases of one general condition requiring varied treatment. The condition is a lapse from health, and the practitioner is called in to restore that lapse. As the dentist is applied to in the several cases enumerated, the physician is called for in others. When the patient suffers from an aching tooth, the dentist is applied to; when from an aching head, he seeks the physician. An alveolar abscess comes under the dentist's hands; an auricular or lumbar abscess under the physician's. Imperfect masticatory machinery is brought to the dentist for repair; deranged or disordered digestive apparatus to the physician. Now in all those ailments which a physician is called to treat, what higher, what different end has he in view, or by what other motive is he influenced than that which the dentist has in view, and by which he is influenced? The end of both is one. The governing motive of both is one—*the relief of human suffering and the promotion of human health.*

I have said that this is the aim of dentistry; this position, I venture to believe, will be endorsed by my professional brethren. That is my standpoint.

I venture to believe, also, that the advanced step I have taken, in my consideration, will also be conceded, viz.: that the end and aim of the physician, as exemplified in the instances cited, are identical with the end and aim of the dentist. In motive and purpose thus far they are parallel.

Advancing along the line of analysis I come next, most naturally, to the allied fact (allied to what has just been urged, indeed, absolutely inseparable from it), that as the medical doctor requires education in order to be competent to treat a prolonged headache, or an auricular abscess, or an impaired digestive apparatus, so does the dentist require education to fit him to treat the diseases of the teeth and contiguous parts which come under his care. And what kind of education? The same in kind as does the physician. Although the region in which his operations are confined is small when compared with the whole human frame, yet within it prevail the same laws, exists the same complex machinery, is found the same liability to derangement, the same need of the same treatment, and the same susceptibility to the same influences that are found elsewhere in the human organism, except that in this region they are more obscure, hence more difficult to understand, than in many other parts of the human body.

To fit him for his work as a dentist, in the full signification of that term as defined by the author of the paper under consideration, it is necessary that a man should have a knowledge of the constituent parts of the oral cavity and their physical relation to each other, viz.: the bones—their shape, size, structure, situation and union; the blood-vessels—their situation, comparative importance, connections and character; the nerves—their origin, connections, ramifications. The branch of study in which this knowledge is gained is called Anatomy.

It is quite necessary, also, that he understand the functions of these various organs and constituent parts; what they are expected to do when in health, and what effect upon adjacent or remote tissues or organs their impairment is likely to produce; to understand that the entire nervous system, for instance, may be thrown into disorder by a lesion in one remote part of it, and consequences the most serious be the result. Although not a gynæcologist, he should know that injudicious operations upon the teeth of a pregnant woman, may, and often do, result in miscarriage; that neuralgia may often be cornered and cured by certain operations upon the teeth, which, but for such intimate knowledge of the nerves and their relations and their influence, would never have been thought of. And that neuralgia may follow as the result of operations unskillfully performed, or ill-timed, or from methods unwisely employed. Here is a branch of study extensive enough for all one's time and all one's energies. It is called Physiology. Into it, to a certain extent, our perfect dentist must enter. And further, it is quite as necessary when the dentist meets with a disease in the region under his care, and locates it, and determines the character and extent of it, and is impressed with the importance and possible consequences of its continuance, I say it is quite as necessary that he know how to treat it, what remedies to employ and how and when to employ them. And if he is to be the ideal dentist described by the author on the table, he should, nay, he must know whether the treatment indicated is to be topical or systemic, surgical or pharmaceutical, palliative or radical; and when he selects either, he must know why he chooses it rather than the others; he must know what to look for as the result of its employment, and when its further use is to be discontinued. What is this but Therapeutics ? Limited, yes ! *but therapeutics still.*

The anatomy of the mouth and contiguous parts is regional anatomy, but none the less anatomy. The physiology that concerns the dentist's field of operations may be called limited, but it is, nevertheless, physiology, and not so limited, either, as one might at first suppose.

And now it is fair, and it is time, to ask further—which of the learned professions requires, as a condition of fitness in its practitioners, a knowledge of anatomy and physiology and therapeutics ? Does Divinity ? Does law ? Nay, but medicine does. And we have seen that dentistry does. It is superfluous for me to say, note the kinship. Your intelligence, gentlemen, has anticipated me, and drawn its own inevitable inference from the line of thought pursued.

The healing art is the parent of all systems of treatment of disease in the human body, whether it be an ingrowing toe-nail, a defective tooth, a diseased eye, or a disordered brain.

Even the "old farmer," in Dr. Kingsley's homely and not at all pertinent illustration, if by close observation or some study he had learned when and how to "open the boil, for one of his laborers, to let out the core," was, to just that degree, practicing the healing art. Nor should his crude but honest efforts to relieve human suffering subject him to ridicule, or be used to point a slur at a body of respectable gentlemen engaged in a respectable vocation. What would be the first exclamation the laborer or his friend would be likely to make after the "old farmer" had completed the operation and shut up the jack-knife which he had used in doing it ? "Why, neighbor, you're quite a doctor." The recognition of the healing art as the parent of all efforts to relieve physical pain or cure physical ills, is immediate, and with all classes, learned or unlearned. And dentistry, as an organized system of treatment for some of the ills of the human body, is a legitimate child of that recognized parent, bearing upon it birth-marks, displaying characteristics that establish its relationship and make its denial of its parentage an absurd exhibition of pitiful and unbecoming pride, a pride that has no basis and is very belittling to its possessor.

Because dentistry is a specialty, it is none the less a profession. What has thus far been urged as to its kinship to medicine is all equally in support of its title to the name—"profession." An offshoot from general medicine, it must be a profession if medicine is a profession. The same sap flows in the branch that flows in the trunk, and though the branch should grow to dimensions exceeding those of the trunk, it would still draw its sap from that same trunk, would still possess the same qualities, bear and manifest the same nature.

Dentistry is a profession, not because "it is a vocation of beneficence." The work of the societies for the prevention of cruelty to animals and children is a vocation of beneficence. Dentistry is a profession, not because of "universal acknowledgment." Even universal acknowledgment would not make it a profession, and the writer in the same paper has already drawn the life-blood from this argument before it was presented, by saying that the medical fraternity assert that "dentistry is only a mechanical trade." Where does the "universal acknowledgment" come in?

I maintain that dentistry is a profession because of *its relation to medicine*; not because of its relation to "any art from plumbing to sculpture," which may be thought to "have its prototype" within its pale of operations. And what makes medicine—the parent—a profession, the same makes dentistry—the child, the specialty—a profession. Any gentleman can determine for himself what that is.

This proposition is not weakened by the assertion of the author that "the majority of dentists, even if they have the medical degree" cannot treat "the diseases of the family" as well as any "old housewife in the country." This is another illustration which, neither for its pertinency nor its elegance, finds any appropriate place in an argumentative paper before a society of intelligent professional gentlemen.

What specialist, pray, practices general medicine? What dentist pretends, or is expected, to "treat the diseases of the family?" And why should the term "an old housewife in the country" be used to signify an uncultivated person of whom nothing worthy can naturally be expected? Is the country necessarily a place of the direst ignorance? Is the housewife, perforce, a mere scullion? Is it any reproach to a housewife to be old? And if, perchance, she be a "housewife," and be "old," and live "in the country," does she become thereby an appropriate foil to set off the incompetence of dentists to treat the diseases of the family? As well use a stage driver, who can oil an axle or fit a linch-pin, as a comparison to show the unfitness of a wheelwright to decorate the exterior of a coach with a paint brush.

Having established the relationship in point of motive, of end, of preparation, of nature, between dentistry and medicine (to my own satisfaction at least), and having built up something on which to hang a "therefore," I am prepared to reaffirn an assertion I have before made, that dentistry is a specialty of medicine. And having asserted this, which, I think, has been quite conclusively proved, I am now quite ready to go further and assert that dentistry is *more* than a specialty of medicine. It has widened its field. It has employed methods which, belonging to the mechanical arts, are still necessary to the execution of its purposes, its professional purposes. And when a dentist sees an object which he wishes to accomplish for his patient's good, whether it be reducing an irregularity in the teeth, covering with a plate an opening in the roof of the mouth produced by malignant disease, supplying lost teeth, elongating a partially erupted bicuspid, or constructing and hanging *in situ* an artificial velum, if he can sit down in his laboratory and with his own fingers make the necessary appliance for the accomplishment of the object, he is by just so much, the better furnished dentist, and not a whit less professional than if he had conceived the idea but was obliged to get some outside mechanic to do the work.

But, while this is true, it is not his mechanical skill which makes him a professional man. It is the knowledge behind the actual exhibition of the skill. It is the ability to originate the conception which is to be executed. It is the knowledge of the underlying principles which are involved in the case under treatment. It is the understanding of the relation between the part affected and the whole organism, and the probable effect upon the whole of an operation upon that part, as gained by an acquaintance with the laws of physical health, of function, of adaptation, of healing. It is the ability to adapt scientifically the specific treatment deemed necessary, so as not to contravene those laws, but to invite their co-operation in securing a good result. This knowledge is acquired in medical schools, or in departments of study borrowed from medical schools, and embodied in the curriculum of dental schools.

There is no difference of opinion between me and the paper as to the fact that dentistry is a profession. What I have already advanced shows that. But this I aver: Just in proportion as we disclaim our relationship to medicine, and with disdainful stroke cut the ties which connect our vocation with the healing art, to that same degree do we cut ourselves off from the title of professional men. Nor will any amount of artistic talent, as displayed in the work of our hands, save us from being considered mechanics, and mechanics only—mechanics of a high order if you please, but mechanics still—and our vocation a trade. Are we ready for this? I, for one, am not.

The man who devotes his life to the manufacture of porcelain teeth, or who constructs artificial dentures according to instructions, and after models furnished by another, and whose attainments are confined to that branch, may do his work most satisfactorily, most artistically, but he is not a professional man. He is a mechanic.

Let it not be thought that I disparage the vocation of a mechanic. By no means. I admire and respect mechanical genius as much as any other man, and whatever of that talent I possess myself, I value most highly. But I am endeavoring to call things by their right names in this paper, and I do not propose, on the one hand, to call a profession a trade, though it include, among other things, that which, pursued alone, would be a trade; nor, on the other hand, to call a trade a profession, though the artisan be a man of more cultivated mind, finer artistic taste and greater manipulative skill, than he who owns a diploma. Let each stand on its own bottom. Neither seeks a conflict. Let us not then force one. Neither can I see any reason or wisdom in boldly antagonizing medicine, nor the fraternity of its practitioners. Why institute an invidious comparison? Why force an unsavory issue? What is to be gained? Nothing but ridicule.

It is no argument in favor of the bold utterance of Dr. Kingsley to say that dentists desire "to be considered medical specialists" that they may "stand well in society," and derive a little "dignity" by reflection, from the noble profession of medicine. Such a statement is an unkind slur upon the dental profession, and in its name I repel it. The competent dentist desires no meretricious dignity and no borrowed standing. He asks the standing, and an acknowledgment of the dignity which of right belongs to him, by virtue of his own worthiness of character. Nor need he ask it. It is accorded. A noble character, lofty attainments, faithfulness to duty, these bring their own recognition. The close student is known to be a student, and respected as a student, and when the products of his study are revealed and seen to be of value to the world, his reward and recognition are sure.

The man who is ambitious of fame, if he is willing to work for the good of man, or the advancement of science, need not seek to connect himself with an already honored vocation, to secure it. It will come to him; it will find him out. Such fame is valuable, more so than that fame, or rather notoriety, which comes to a man because of some conspicuous position of antagonism in which he has placed himself.

Neither the incompetent hanger-on to a respected vocation, nor the opinionated declaimer of his independence of it, can hope for permanent fame or sincere regard. The flimsy pretense of the former and the alarming state of inflation of the latter, will each be understood. The enlightened public does not affect either parasites or iconoclasts. I say this in no invidious sense; I speak what all may recognize to be the truth.

The question of participating in the International Medical Congress, to be held in this country during the present year, is disposed of in the paper under review in a few curt sentences, breathing a spirit and a conceit which, to every unprejudiced reader, must seem out of place, unbecoming and uncalled for. What offense has been put upon dentistry by an invitation "to form a section of that Congress," it would be difficult to name; and, indeed, nothing but the diseased eye of jealously or the hypersensitiveness of overgrown pride could perceive any. Instead of an offense it seems a very proper courtesy, and if we are gentlemen, and accustomed to the courtesies of life, it becomes us to maintain that character by meeting courtesy with courtesy.

In considering a question like this, it is well to lay aside any private enmities or prejudices which might warp our judgment and divert our thoughts from matters to men. We may not just relish the form of the invitation, or the men named for prominent positions on committees, or some other detail, but as gentlemen, as professional gentlemen, as members of a profession for which is claimed such exalted eminence, we should not be affected by such trifles, and on their account refuse our co-operation. Much less should we return sarcasm and bitterness for recognition and courtesy. It does not look well, and I am unwilling that our profession should be amenable to the charge of maintaining so ungracious a bearing, through the agency of one of its members, without a protest. It may be, and it may not be deemed best, to go into this Medical Congress. That is quite a distinct matter, to be decided wisely, and after all the circumstances have been well considered. As to our having "no business there" as "an independent profession," I quite agree with that statement. We have no business to go there boastfully assuming that we are an independent profession, and so placing ourselves in direct antagonism with our host.

That we should be out of place, as dentists, depends on circumstances. If we cannot, as dentists, enjoy the society of cultivated men, we should be out of place. If we, as dentists, do not know how to conduct ourselves in such society, we should be out of place. If we are so filled with self-importance that nothing but what we say is considered of any value, then we should be out of place, for we shall meet many gentlemen there who do not think so. But if we are gentlemen, if we realize the aim and character and importance of our vocation as one among the systems whose end and aim are the promotion of human health, if we are possessed of enlightened common sense, then we shall not be out of place when we take the position which they who invite us think it right for us to occupy.

Of one thing, however, we may be assured; if a section of dentistry is formed in that Congress, any credit that shall come from it to our profession will come because it is deserved, and not by favor of the medical fraternity. They will not commend what is worthy or unworthy of commendation, simply because it is connected with their Congress. Medical men are men of judgment and men of honor. If a thing is good they know it and will say so, and if it is bad they know it and will not pronounce it good. Therefore, a dental section in a Medical Congress will stand on precisely the same basis as if it stood alone, viz.:—on its merits, and on these only. There is no need of fearing that our profession will receive a less meed of praise than it deserves, nor that its extraordinary attainments will be used to lift the parent art, medicine, to a loftier altitude. Oh, no! the most conservative gentleman need have no alarm. We shall stand just where we belong, or if we fly at all, it will not be by hitching on to anyone else's kite, but because our own is so well shaped, and so nicely balanced, that soaring is as natural to it as to the even-winged bird.

If the prominent gentlemen to whom this matter of a dental section has been entrusted shall find time in their busy lives to create and equip it, though I may not be able to assist them in person, I shall wish them every success, and shall feel confident that under their wise and efficient management the section will be a credit, both to the Congress of which it shall be a part, and to the profession which it shall represent.

As to an International Dental Congress, if in the future it shall seem wise to convene such a body, and the motive be the interchange of matured views, the revelation of discovered principles, the honest discussion of methods, the introduction of new ideas, all to the end of promoting greater efficiency, of affording mutual instruction, mutual encouragement, and as a result, of attaining an advanced position for dentistry, then I shall raise no objection to it, for the motive will be worthy and the end desirable. But if the chief aim of such a Congress shall be to attract to ourselves "the eyes of the whole world," while we spend our time and breath in praising and admiring ourselves, then I shall be opposed to it as undignified, and unworthy of professional gentlemen.

Let dentists honor their profession; it is worthy of it. Let them advance along the lines of investigation as far as they can. Let them bend all their energies to the end of making every detail in operation reach the highest degree of excellence possible. Let them study, experiment, analyze, that occult things may be brought to light; that hidden laws may be revealed; that obscure relations may be understood; that greater efficiency in substituting artificial for natural organs may be attained; that the lines of beauty in the human face may be more perfectly preserved. Let them place their ideal high, nor be content till, in place of a haggard, sunken countenance, a toothless mouth, diseased gums and impaired health, brought into our offices by their unhappy possessors, there may go out therefrom a well-rounded face, whose features shall have been restored to symmetrical outline; a well-ordered set of masticating organs, their naturalness or artificialness known only to the possessor and the dentist; a healthy mouth, everything in order for the proper preparation of the food and for the production of the quickly succeeding condition of good digestion; cheerful spirits; in a word, good heath.

Let dentists strive for such ends, and they will not only be useful, but noble men; they will not only attain eminence and elevate their vocation, but they will find their heads and their hands so full of thought and act that there will be no time, as there will be no need, to look around for "recognition," for "standing," for confession of "dignity." There will be no time, as there will be no need, to argue upon the claim of dentistry to the name of a profession. There will be no time, as there will be no need, to search for a balance which will exalt dentistry above medicine when they are weighed against each other. There will be no time, as there will be no need, to labor for the creation of a board of the eru'dite of the land, who shall by formal edict, proclaim to all the earth that henceforth dentistry is a distinct profession, disconnected with and absolutely independent of every other.

When the ultimate limit of the capacity of this generation shall have been reached, and our successors, taking up the science of dentistry where we leave it, shall carry it on to degrees of efficiency now unrealized, and exhaust their powers upon it till it stands a wonderful product of active, human intelligence, a marvel in its resources, its achievements, its beneficent possibilities, even then, inevitably then, unhesitatingly then, will the keen eye of the impartial critic detect the unbroken line of connection, the vital, umbilical cord, which, running back through all the variations of direction and apparent diversity of operation, shall be seen to be attached inseparably and forever to the healing art as its legitimate parent, and the instant verdict shall be—THIS IS A SPECIALTY OF MEDICINE.

